# Direct microfluidic antibiotic resistance testing in urine with smartphone capture: significant variation in sample matrix interference between individual human urine samples†

**DOI:** 10.1039/d1ra06867a

**Published:** 2021-11-29

**Authors:** Sarah Helen Needs, Sultan İlayda Dönmez, Alexander Daniel Edwards

**Affiliations:** School of Pharmacy, University of Reading UK a.d.edwards@reading.ac.uk s.h.needs@reading.ac.uk

## Abstract

Rapid and portable direct tests for antibiotic resistance in human clinical samples such as urine could reduce misuse of precious antimicrobials, by allowing treatment decisions to be informed by microfluidic diagnostic tests. We demonstrate that the variable composition of human urine can significantly affect the antibiotic minimum inhibitory concentration (MIC) measured using microfluidic devices. The urine sample matrix interference was not observed in pooled normal urine, emphasising the critical importance of assessing matrix interference with a wide range of individual urine samples, rather than a few standardised or pooled controls. Both dilution into assay medium and inclusion of buffer could reduce the matrix interference, but dilution may affect analytical sensitivity by increasing the minimum bacterial cell density needed in a sample for growth to be detected, especially for miniaturised devices that test small sample volumes. We conclude it is vital to fully assess and optimise novel analytical microbiology tools using multiple individual urine samples, otherwise the high variation in matrix interference will compromise the clinical performance of these rapid diagnostics that are urgently needed to tackle the global threat of antimicrobial resistance.

## Abstract 2

Direct antibiotic susceptibility tests for urinary tract infections should be assessed using a wide range of individual urine samples to identify matrix interference.

## Graphical abstract

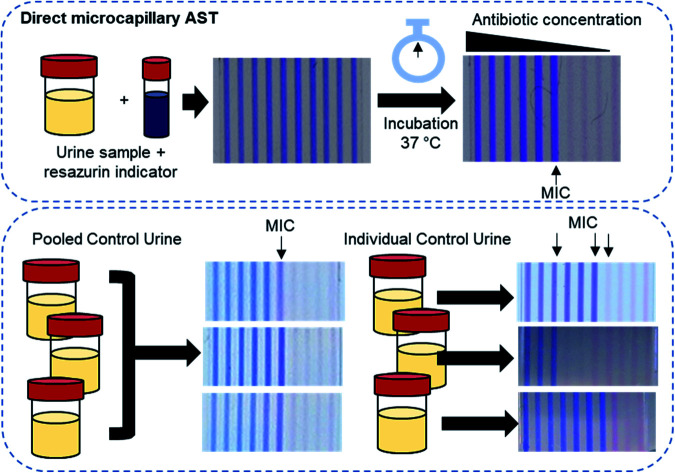

## Introduction

Improved diagnostics and rapid testing is vital to minimise mis- and overuse of antibiotics to reduce the global threat of antimicrobial resistance (AMR),^1,2^ and miniaturised analytical technology is emerging to achieve this.^3^ Urinary tract infection (UTI) is a widespread clinical need where initial antibiotic selection is not routinely guided by antibiotic susceptibility tests (AST), and instead surveillance information is used to develop empirical guidelines to minimise risk of resistance.^4^ Microfluidics offer portability, speed, and ease of use compared to traditional laboratory microbiology methods, including broth microdilution and disc diffusion, which take at least 48 h from sample collection. Whilst molecular methods and other emerging analytical microbiology tools are increasingly able to rapidly detect certain pathogens or resistance genes, phenotypical AST remains vital to measure pathogen response to antibiotic functionally, and for this reason remains the clinical reference standard.

Significant time could be saved if clinical samples can be tested directly, avoiding overnight plating and colony isolation. Many of the emerging novel and microfluidic devices for rapid phenotypic AST determination use urine as the clinical sample. UTI represent a sample with a high clinical need and a high bacterial load, allowing direct testing without bacteria enrichment. However, although urine matrix components are known to be able to affect analytical performance of established functional microbiology tests,^5^ this has not been extensively studied in the emerging microfluidic microbiology tools.

Some recently described methods remove the sample matrix effect by using bacteria traps or filters to exchange the urine with growth media.^6–8^ However, these methods add further liquid handling steps. Other systems dilute the sample by at least 1 : 2 (ref. 9) but usually more, by 1 : 10.^10,11^ While many novel microfluidic microbiology devices have been recently published that deliver AST results, few of these have been validated with large numbers of normal human urine or clinical samples from patients and antibiotic combinations (Table S1†).

Here we present data examining the impact of variation in urine matrix composition on MIC measured in a low-cost microcapillary device, based on simple disposable microcapillary arrays. The test strips are a miniaturised version of the clinical reference standard microplate broth microdilution (BMD) method. Each test strip has 10 parallel capillaries, each of which is analogous to one well of a microplate, simplifying operation and increasing throughput compared to microplates. The microcapillary strips are mass-produced by melt-extrusion, allowing high volume of the AST strips to be made in comparison to other experimental microfluidic systems (Fig. S1†). The results of the AST are readable by both smartphone and robotic timelapse imaging, ideal for systematic analysis of matrix interference in microfluidic microbiology assays. Having previously established proof-of-concept of microbial identification, viable cell counting, and monitoring bacteriophage lysis^12–15^ in this system, we have not previously used the system to interrogate the impact of urine sample matrix on microfluidic antibiotic resistance assays. Here we assessed in detail the impact of urine matrix on microfluidic AST, by systematically quantifying minimum inhibitory concentration (MIC) in the presence or absence of a wide range of human urine samples. For direct testing of clinical urine samples to be successful with microfluidic versions of current clinical reference standard (*e.g.* microplate broth microdilution), a full understanding of urine matrix interference is essential. Of particular note, we evaluated the impact of urine on performance using a panel of individual urines *vs.* pooled controls. The mass-produced nature of the tests allowed greater than >2000 urine/isolate/antibiotic combinations to be analysed, with full growth kinetics monitored in every condition. Our findings are applicable to many other microfluidic researchers developing direct AST systems for urinary tract infections.

## Results and discussion

**Fig. 1:**
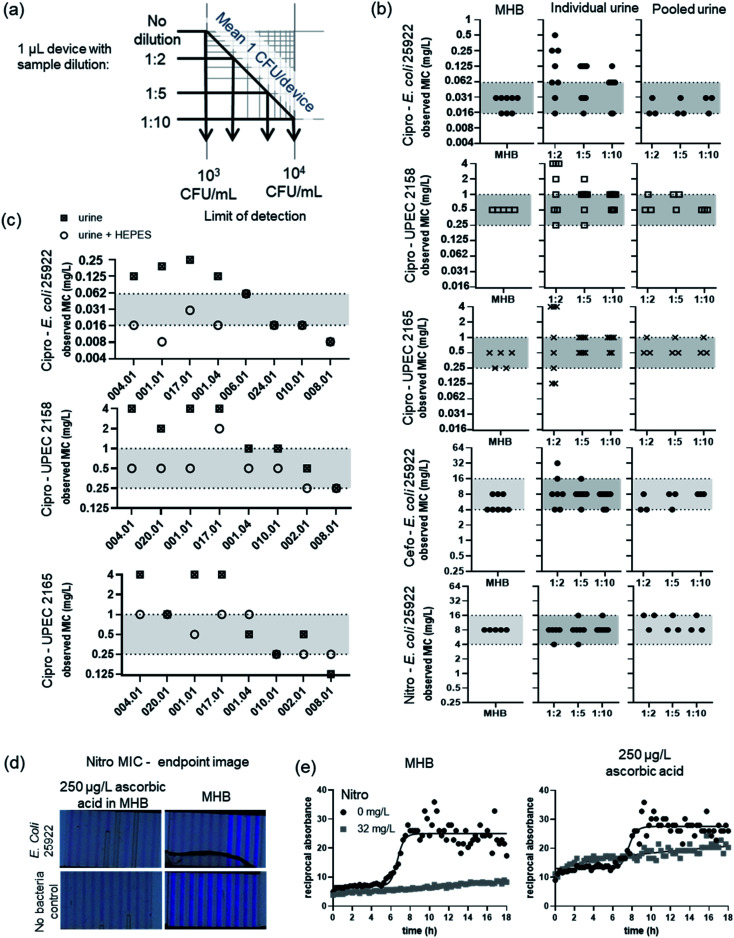
Variation in AST measurements in individual urine samples. (a) Dilution of sample can reduce inter sample variation but decreases limit of detection. (b) Variation in MIC for ciprofloxacin for *E. coli* 25922 QC strain and 2 uropathogenic *E. coli* strains 2158 and 2165. MIC performed for *E. coli* 25922 for cefoxitin and nitrofurantoin. MIC performed in Mueller–Hinton broth, 7 individual healthy urine samples or 3 pooled urine samples. Horizontal grey area indicates the highest MIC determined in MHB ± 1 log2 antibiotic dilution (c) MIC for ciprofloxacin performed in individual urine samples diluted 1 : 2 for isolates 25922, 2158 and 2165 with and without 10 mM HEPES. Urine samples are sorted from left to right with lowest pH to highest. pH ranges from 6.44–8.42 (d) images of nitrofurantoin MIC microcapillary BMD tests after 18 h incubation. (e) Timelapse resazurin colour change of *E. coli* 25922 grown in the absence and presence of 32 mg L^−1^ nitrofurantoin in MHB or MHB supplemented with 250 μg L^−1^ ascorbic acid. Solid line indicates 4 parameter logistic curve.

One of the simplest ways to eliminate matrix interference in any analytical technique is dilution, which both reduces the concentration of interfering agents, and provides an opportunity to mitigate interference for example by buffering. However, dilution decreases analyte concentration, potentially compromising clinical measurements if the limit of detection no longer matches the clinical threshold. For AST, the ‘analyte’ is pathogenic bacteria, and in UTI the clinical threshold is 10^5^ colony forming units (CFU) per mL.^16^ This allows us to calculate an absolute threshold for measuring antibiotic susceptibility such that ≥1 CFU is present per test, for different urine dilutions, in small sample volumes. Microfluidic device design varies considerably, so test volumes vary from picolitres to mL.^7,11,17^ However, for microdevices having test volumes below 1 μL, high dilution is likely to compromise utility for clinical UTI samples. The distribution of bacteria in microfluidic devices is often dictated by Poisson statistics.^12,18^ Even if one single CFU can be detected, the limit of pathogen detection, where 99% of the time a 1 μL device will contain at least 1 bacterium, is 5 × 10^3^ CFU mL^−1^; a 1 : 10 sample dilution raises this to 5 × 10^4^ CFU mL^−1^ (Fig. 1a). Fewer cells can also make detection slower.^12^ We conclude that urine matrix interference becomes an especially important consideration for microfluidic microbiology, given this connection between sample volume and limit of detection, compared to established microplates or agar Petri dishes assays where sample and assay volumes are larger.

Antibiotic sensitivity can be quantified by measurement of MIC; the MIC can be compared to standards, reference strains and internationally agreed breakpoint antibiotic concentration to score resistance *vs.* sensitivity. We therefore explored how urine matrix affects the measured MIC in microfluidics, compared to controls without adding urine. The MIC was determined for one quality control strain (*E. coli* ATCC 25922) and two uropathogenic *E. coli* isolates, spiked into human urine: firstly with pooled control urine; and secondly with a panel of individual urine samples. Even when diluted by only 1 : 2 in growth detection media, the observed MIC in the presence of pooled urine was within the reference range, differing by a maximum of 1 doubling dilution of antibiotic, for three important UTI antibiotics (Fig. 1b and S2†).

However, a very different picture emerged with individual urine samples. Whilst observed MIC for QC strain 25922 for nitrofurantoin were within the reference range for all urines, significant variation in observed MIC appeared for cefoxitin, and 1 out of 8 urine samples diluted at 1 : 2 gave an observed MIC 1 doubling dilution outside the reference range. The urine matrix interference was greatest with ciprofloxacin, with the observed MIC ranging over 6 doubling dilutions. This high variation in observed MIC was consistent in all strains tested. The breakpoint for ciprofloxacin is 0.25 mg L^−1^, indicating that if tested in a range of normal human urines, 12.5% (1/8) of the urine samples containing *E. coli* 25922 would be miscategorised as resistant. The MIC of the UPEC isolates was too close to the breakpoint to categorise (resistant or susceptible), but this high variation in observed MIC suggests that individual urines contain matrix components that can significantly interfere with microfluidic AST.

Urine is a complex mixture of different components and can vary significantly between and within patient samples depending on diet and hydration, time of collection, and age, among other factors. We investigated the different elements of urine matrix that might interfere with microfluidic AST using our panel of human urine samples spiked with bacteria. We found that urine pH accounted for a significant portion, but not all of the variation of observed MIC. The urine samples that changed MIC the most were the most acidic, all having pH lower than 7 (Fig. 1c, d and Table S2†).

The activity of several antibiotics can be influenced by pH, possibly explaining the impact of urine pH variation on *in vitro* tests. For example ciprofloxacin, meropenem, trimethoprim, fosfomycin, amikacin, colistin and ertapenem have higher observed MIC values in acidic media.^19–21^ An acid environment lowered the MIC of nitrofurantoin indicating an acid environment increases efficacy.^22^

To establish how much of the interference by urine matrix could be attributed to pH variation, we added 10 mM HEPES to urine samples by inclusion in growth indicator medium. Buffering decreased the variation in observed MIC between individual urines, such that with HEPES 83% (20/24 urine sample/isolate combinations) were within the reference range in contrast to only 46% without buffer (11/24) (Fig. 1c). This demonstrates that pH is a major source of urine matrix interference on microfluidic AST, but does not account for all the variation.

To achieve the fastest AST results, minimising time to detection is an important target. As well as influencing antibiotic activity, urine matrix can interfere with growth kinetics. High levels of urine can increase the time for bacterial detection, with pH being a significant factor.^23^ Analysis of growth kinetics can deliver faster results than endpoint measurements: for fast growing *E. coli* this can cut time from overnight (endpoint) to just a few hours.^12^ Kinetic analysis revealed that the addition of HEPES to growth media into which urine sample 001.01 was diluted, achieved the same MIC result as that of the MHB control, and with the same rapid time to detection. Bacteria were detected at 4.2 h for MHB, 4.3 h for urine with HEPES and 4.8 h for urine without buffer (Fig. S3†). This indicates that the variation in pH of urine samples can delay microfluidic bacterial growth detection, as well as measurement of antibiotic MIC.

Ascorbic acid is found at variable concentration in urine. There is already significant evidence of ascorbic acid interference with POC urine dipstick tests.^24,25^ Ascorbic acid can also interfere with phenotypic tests that use resazurin as growth indicator, as it can reduce the dye even in the absence of bacteria.^26^ Here, we found concentrations above 125 μg L^−1^ ascorbic acid in MHB lead to a significant decrease in the colour of resazurin in microdevices, levels that were exceeded in ascorbic acid tests on the panel of individual urine samples (Table S2†). This steady decrease in resazurin associated with high ascorbic acid levels could be mistaken for bacterial growth if a simple endpoint readout is used (Fig. 1d). A loss of blue colour is seen at 250 μg L^−1^ ascorbic acid even in the absence of bacteria and MIC cannot be determined. Timelapse imaging has been used in a number of novel rapid AST systems including microfluidic devices, as it allows the earliest time point for bacterial growth detection to be used for fastest results. Here, we show that kinetic analysis has a further benefit, allowing discrimination between characteristic exponential changes following bacterial growth, from linear changes associated with interfering factors such as high levels of ascorbic acid in urine sample matrix. If using a single measurement timepoint, it is difficult to distinguish growth in samples with ascorbic acid levels, because the starting colour of resazurin can be reduced in the absence of bacteria, with control sample colour being similar to that seen after bacterial growth. Kinetic growth analysis of a nitrofurantoin microcapillary BMD revealed clear evidence of bacterial growth (s-curve) in contrast to the steady linear change indicating no growth at a concentration of nitrofurantoin that inhibits bacterial growth, even in the presence of 250 μg L^−1^ ascorbic acid – higher than found in any individual urine sample (Fig. 1e). At concentrations of ascorbic acid that affected the colour change of resazurin there was still no significant difference in MIC for nitrofurantoin or ciprofloxacin (Fig. S4†).

Urea is the most abundant chemical in urine; when we included up 20 mg mL^−1^ urea in artificial urine no effect was found on resazurin indicator. Increasing urea concentration only started to affect MIC measurement for nitrofurantoin at 60 mg mL^−1^, and the MIC for ciprofloxacin remained within the expected range up to 60 mg mL^−1^ urea (Fig. S4†). This suggests that urea is unlikely to interfere in microfluidic AST.

## Conclusion

We conclude that microfluidic microbiology systems developed to test urine should be optimised using multiple individual urine samples, as pooling urine to make controls removes the high level of variation in composition. For resazurin-based growth measurements in microsystems, variation in pH appears to have the biggest impact on accuracy of antibiotic susceptibility testing, with higher levels of ascorbic acid found in some urine samples being particularly problematic for colorimetric endpoint assays. Kinetic growth analysis can partly overcome this interference, and offers improvement over endpoint growth estimation. Adding buffer to the growth medium reduces but does not completely eliminate variation. Higher dilutions of urine can reduce or eliminate matrix interference, at the cost of slower growth detection and with implications for microfluidic device design, especially the lower limit of sample volume able to detect microbes in clinical samples with lower pathogen cell concentrations.^12^ Furthermore, for isolates with a large difference between MIC and breakpoint, using only a single antibiotic concentration to determine susceptibility/resistance can miss this variation, minimum inhibitory concentration for some samples should be evaluated.

The potential for microfluidics to address the global challenge of AMR can only be realised if careful attention is paid to the analytical challenge of clinical samples. The panel of individual urine samples assessed in this article was taken from healthy volunteers, and clinical samples from patients presenting with urinary tract infection are likely to be more complex and with higher variation in matrix composition. Further pathological changes such as patient cells present in the sample (red blood cells and leukocytes) may also cause further interference in novel AST devices. However, the factors described here will certainly vary in clinical samples and this key insight into individual variation in urine matrix interference on microfluidic microbial measurement will also relate to other direct AST systems. Validating and optimising novel assays in pooled or synthetic urine is likely to lead to analytical problems arising in real samples where higher variation in composition can be expected.

## Experimental section

### Urine samples and isolates

Ethical consent for the collection of urine from healthy donors was received from the University of Reading, reference code 19/59. Informed written consent was obtained from all participants. Urine samples were collected in Brand Urine beakers under instruction to collect a midstream urine sample. Samples were collected and tested within 4 h using Uritest 10V Urinalysis strips and Quantofix Ascorbic Acid test (Sigma Aldrich, UK). pH was determined by pH electrode. Following this urine samples were filtered using a 5 μm syringe filter and stored at −20 °C until use. *E. coli* 25922 was purchased from ATCC. Uropathogenic *E. coli* (UPEC) isolates 2158 and 2165 were collected at a tertiary care hospital of Pakistan from community acquired UTI patients.^27,28^ Ethical approval was obtained from the ethical review board of the Pakistan Institute of Medical Sciences.

### Microcapillary broth microdilution

Microfluidic MIC test strips were prepared as described.^13^ Briefly, microcapillary film with 10 parallel capillaries of 270 μm capillary diameter were hydrophilic coated by incubating 5 mg mL^−1^ polyvinyl alcohol (PVOH) (*M*_w_ 146, 000–186, 000, >99% hydrolysed, Sigma Aldrich, UK) for 2 h at room temperature. The PVOH was removed by washing the capillaries with sterile Ultrapure Milli-Q water. Antibiotics at the concentration indicated were injected into individual capillaries, frozen at −80 °C for >1 h and freeze-dried for >4 h on an Edwards Modulyo freezer-drier. Test strips were vacuum sealed and stored at −20 °C until use for no longer than 1 week.

*E. coli* were grown in MHB and diluted to 5 × 10^5^ CFU mL^−1^ in urine and MHB with resazurin indicator medium to a final concentration of 0.25 mg mL^−1^. Samples were loaded into the microfluidic test strips by adding 200 μL to a well of a 96 well plate and dipping the test strip into the well. The sample is taken up into the capillaries by capillary action. Each end of the test were sealed with silicone grease and incubated overnight at 37 °C. MIC was recorded using iPhone 6s or Canon Powershot S120 and scored by eye based on colour change of resazurin from blue to pink or recorded on an imaging robot.^29^

### Microplate broth microdilution

*E. coli* were grown in MHB and diluted to 0.5 McFarland standard equivalent. Bacteria isolates were then diluted 1 : 100 in MHB and 50 μL were added to microplate wells. Microplate wells already contained 50 μL of antibiotic solutions and resazurin. The final concentration of the wells contained doubling dilutions of antibiotics, resazurin dye at 0.25 mg mL^−1^ and bacteria at 5 × 10^5^ CFU mL^−1^. Plates were incubated overnight at 37 °C. MIC was determined by the lowest concentration of antibiotic in which the resazurin remained blue.

## Funding

This research was supported by the EPSRC (EP/R022410/1).

## Ethical approval

The collection of urinary pathogenic *E. coli* from a tertiary care hospital of Pakistan from community acquired UTI patients and was approved by Ethical Review Board (ERB) of Pakistan Institute of Medical Sciences. Ethical consent for the collection of urine from healthy donors was received from the University of Reading, reference code 19/59. Informed written consent was obtained from all participants.

## Conflicts of interest

ADE is one the inventors of patent application protecting aspects of the novel microfluidic devices tested in this study and is a director and shareholder in Capillary Film Technology Ltd, a company holding a commercial license to this patent application: WO2016012778 “Capillary assay device with internal hydrophilic coating” AD Edwards, NM Reis.

## Supplementary Material

RA-011-D1RA06867A-s001
