# Supplementary material for: Direct microfluidic antibiotic resistance testing in urine with smartphone capture: significant variation in sample matrix interference between individual human urine samples
Source: RSC Adv. 2021 Nov 29;11(60):38258–63. doi: 10.1039/d1ra06867a (PMC9044048; doi:10.1039/d1ra06867a)
Supplement: RA-011-D1RA06867A-s001 [file RA-011-D1RA06867A-s001.pdf]

Electronic Supplementary Material (ESI) for RSC Advances.
This journal is © The Royal Society of Chemistry 2021

Supporting Information

Direct microfluidic antibiotic resistance testing in urine with smartphone capture: significant variation in sample matrix interference between individual human urine samples

Sarah H Needs[1], Sultan İlayda Dönmez[1] and Alexander Daniel Edwards[1]
[1]School of Pharmacy, University of Reading, UK

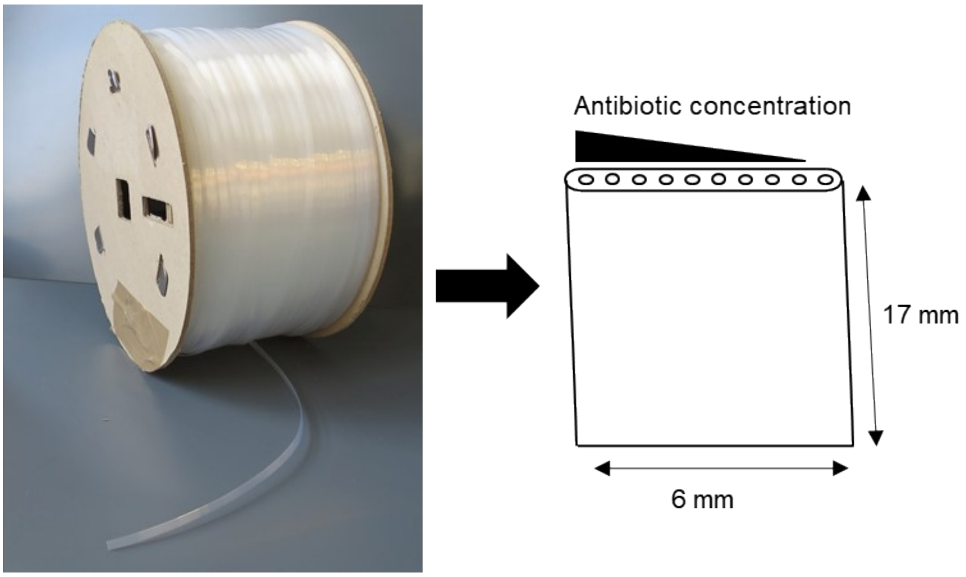

Figure S1. Large batches of microcapillary film are produced by melt-extrusion and transformed into miniaturised broth microdilution ‘dip and test’ strips

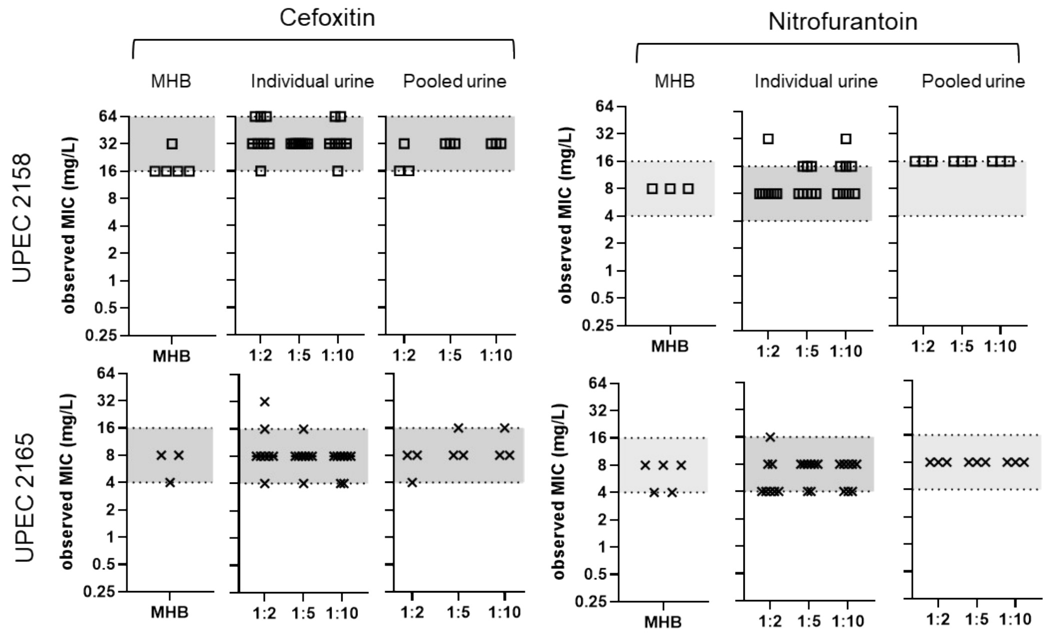

Figure S2. Minimal variation in MIC was observed with cefoxitin and nitrofurantoin for two uropathogenic *E. coli* isolates diluted in urine. *n>3*. Each data points indicate a microcapillary BMD test. For individual urine samples, each data points indicate a different urine sample with at least seven urine samples tested.

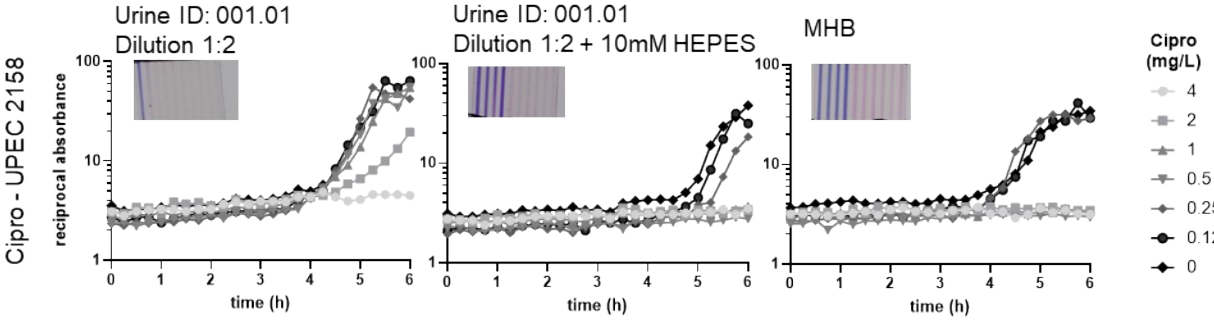

Figure S3. Growth curves of MIC for ciprofloxacin for UPEC 2158 in urine sample 001.01 diluted 1:2 without and with HEPES 10 mM or in MHB alone. Image insert indicates microfluidic test strips with antibiotic concentration left to right: 4,2,1,0.5,0.25,0.125,0.062,0.031,0.016,0 mg/L.

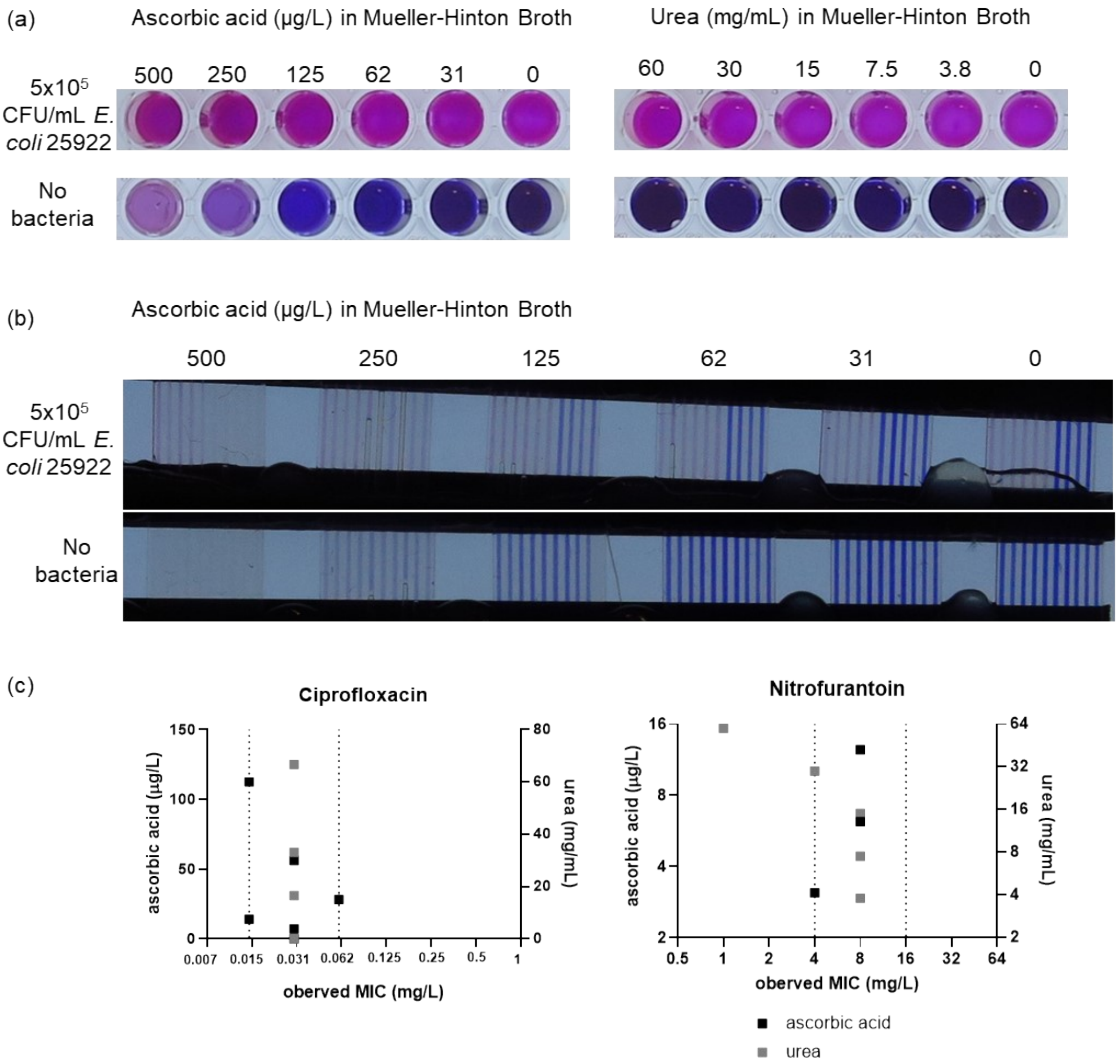

Figure S4. High levels of ascorbic acid interfere in resazurin indicator use. (a) Ascorbic acid and urea were diluted in MHB and inoculated with and without *E. coli* 25922 overnight at 37 °C. (b) Nitrofurantoin MIC test strips ranging from left-right, 0-64 mg/L nitrofurantoin were tested with ascorbic acid diluted in MHB and incubated overnight at 37 °C. (c) The observed MIC results for nitrofurantoin and ciprofloxacin in MHB supplemented with either ascorbic acid or urea performed in microcapillary test strips. Vertical dotted lines indicate ± 1 doubling dilution of antibiotic from the MHB control.

Table S1. Summary of articles using direct urine sampling for antimicrobial susceptibility tests in microfluidic devices

Sample	Summary	MIC or Single antibiotic concentration	Reference
Spiked healthy urine (sample exchanged with media)	Bacteria trap in channels – single cell time lapse imaging	Single concentration	(1)
Spiked healthy urine (commercial urine)	Urine diluted 1:20 and mixed with antibiotics prior to loading in microchannel and electrical signals measured	MIC	(2)
Spiked culture negative urine (sample exchanged with media)	Bacteria growth determined by resazurin fluorescence in the presence or absence of antibiotics in droplets.	Single concentration	(3)
5 positive clinical samples (sample exchanged with media)	Filter bacteria from urine and resuspended in growth media – resazurin detection in nanolitre array	Single concentration	(4)
25 clinical samples diluted 1:10 in media (21 bacteria positive samples)	Single cell trapping and imaging in microchannel and imaging. Sample mixed with breakpoint antibiotic before loading	Single concentration	(5)
24 clinical sample diluted 1:2 in media (12 bacteria positive samples)	Bacteria incubated with antibiotics and single cell counted in cuvettes using light scattering microscopy	MIC	(6)
60 clinical samples diluted 1:10 in media (30 bacteria positive samples)	Bacteria incubated with antibiotics and single cell counted in cuvettes using light scattering microscopy	Single concentration	(7)
215 clinical samples diluted 1:2	Urine incubated with antibiotics for 2.5h followed by electrochemical based 16S rRNA detection	Single concentration	(8)

Table S2. pH and dipstick tests of urine samples

	Urine ID	pH electrode	LEU/µL	NIT (mg/dL)	URO (mg/dL)	PROTEIN (mg/dL)	BLOOD (Ery/µL)	SPECIFIC GRAVITY	KET	BILIRUBEN (mg/dL)	GLUCOSE (mg/dL)	ASCORBIC ACID (µg/L)
1	001.01	6.83	NEG	NEG	0.2	NEG	NEG	1.025	NEG	1	NEG	0
2	004.01	6.44	NEG	NEG	NEG	15	POS (+)	1.015	NEG	1	NEG	0
3	010.01	7.79	NEG	NEG	NEG	15	NEG	1.01	NEG	NEG	NEG	50
4	008.01	8.42	NEG	NEG	NEG	15	POS (+)	1.005	NEG	NEG	NEG	50
5	017.01	6.95	NEG	NEG	NEG	30	NEG	1.03	NEG	NEG	NEG	50
6	002.01	7.95	NEG	NEG	NEG	15	NEG	1.01	NEG	NEG	NEG	100
7	006.01	7.68	NEG	NEG	NEG	30	5-10	1.02	NEG	NEG	NEG	300
8	020.01	6.46	NEG	NEG	NEG	15	POS (+++)	1.015	NEG	NEG	NEG	50
9	001.04	6.97	NEG	NEG	NEG	15	NEG	1.010	NEG	1	NEG	100
10	012.01	8.31	700+ Leu	NEG	NEG	NEG	NEG	1.005	NEG	NEG	NEG	0
11	001.03	7.59	NEG	NEG	NEG	NEG	NEG	1.01	NEG	NEG	NEG	50
12	013.01	8.37	NEG	NEG	NEG	NEG	NEG	1.01	15	NEG	NEG	100
13	024.01	7.75	NEG	NEG	NEG	15	NEG	1	NEG	NEG	NEG	0
14	014.01	7.99	NEG	NEG	NEG	15	NEG	1.005	NEG	NEG	NEG	0
15	015.01	7.65	NEG	NEG	NEG	15	NEG	1.015	NEG	NEG	NEG	100
16	007.01	8.03	NEG	NEG	NEG	NEG	POS (+)	1	NEG	NEG	NEG	0

Table S3. Composition of pooled urine samples

	Number of individual urine samples	Urine ID
Pooled urine 1	5	012.01, 001.03, 013.01, 006.01, 001.04
Pooled urine 2	5	024.01, 008.01, 010.01, 004.01, 002.01
Pooled urine 3	15	012.01, 001.03, 013.01, 006.01, 001.04, 014.01, 024.01, 008.01, 010.01, 004.01, 002.01, 015.01,

		001.01, 007.01

1. Baltekin Ö, Boucharin A, Tano E, Andersson DI, Elf J. Antibiotic susceptibility testing in less than 30 min using direct single-cell imaging. Proceedings of the National Academy of Sciences. 2017;114(34):9170.
2. Yang Y, Gupta K, Ekinci KL. All-electrical monitoring of bacterial antibiotic susceptibility in a microfluidic device. Proceedings of the National Academy of Sciences. 2020;117(20):10639.
3. Zhang P, Kaushik AM, Mach KE, Hsieh K, Liao JC, Wang T-H. Facile syringe filter-enabled bacteria separation, enrichment, and buffer exchange for clinical isolation-free digital detection and characterization of bacterial pathogens in urine. The Analyst. 2021;146(8):2475-83.
4. Avesar J, Rosenfeld D, Truman-Rosentsvit M, Ben-Arye T, Geffen Y, Bercovici M, et al. Rapid phenotypic antimicrobial susceptibility testing using nanoliter arrays. Proceedings of the National Academy of Sciences. 2017;114(29):E5787.
5. Li H, Torab P, Mach KE, Surrette C, England MR, Craft DW, et al. Adaptable microfluidic system for single-cell pathogen classification and antimicrobial susceptibility testing. Proceedings of the National Academy of Sciences. 2019;116(21):10270.
6. Mo M, Yang Y, Zhang F, Jing W, Iriya R, Popovich J, et al. Rapid Antimicrobial Susceptibility Testing of Patient Urine Samples Using Large Volume Free-Solution Light Scattering Microscopy. Analytical chemistry. 2019;91(15):10164-71.
7. Zhang F, Jiang J, McBride M, Yang Y, Mo M, Iriya R, et al. Direct Antimicrobial Susceptibility Testing on Clinical Urine Samples by Optical Tracking of Single Cell Division Events. Small. 2020;16(52):2004148.
8. Mach KE, Mohan R, Baron EJ, Shih M-C, Gau V, Wong PK, et al. A biosensor platform for rapid antimicrobial susceptibility testing directly from clinical samples. The Journal of urology. 2011;185(1):148-53.

References
